# The effects of diet on levels of physical activity during winter in forensic inpatients – A randomized controlled trial

**DOI:** 10.29219/fnr.v64.3610

**Published:** 2020-02-21

**Authors:** Anita L. Hansen, Gina Ambroziak, David Thornton, Lisbeth Dahl, Bjørn Grung

**Affiliations:** 1 Department of Clinical Psychology, University of Bergen, Bergen, Norway and Department of Psychosocial Science, University of Bergen, Bergen, Norway; 2 Centre for Research and Education in Forensic Psychiatry, Haukeland University Hospital, Bergen, Norway; 3 Sand Ridge Secure Treatment Center (SRSTC), Mauston, WI, USA; 4 FASTR, Madison, WI, USA; 5 Institute of Marine Research (IMR), Bergen, Norway; 6 Department of Chemistry, University of Bergen, Bergen, Norway

**Keywords:** Fatty fish consumption, meat consumption, physical activity, mental health problems

## Abstract

**Background:**

Fish consumption has been shown to have beneficial effects on biological and subjective measures of health and well-being. However, little is known about the effects of fish consumption at the behavioral level.

**Objective:**

The primary aim of this study was to investigate the influence of diet on behavior such as physical activity during winter in forensic inpatients. The secondary aim was to investigate the relationship between vitamin D status and physical activity.

**Design:**

Eighty-one male forensic inpatients participated in this study. Participants were randomized into two different diet groups: a Fish group receiving fatty fish three times per week and a Control group receiving an alternative meal (e.g. chicken, pork, and beef); while the Fish group received their fish, the Control group received an alternate meal, but with the same nutritional value as their habitual diet. The duration of the food intervention was 6 months.

**Results:**

The results revealed that the Fish group had a regular pattern of physical activity throughout the intervention period. The participants in the Control group showed a more irregular pattern of physical activity in addition to a significant reduction in physical activity over time.

**Conclusion:**

Behavior such as physical activity during winter seemed to be influenced by the diet.

## Popular scientific summary

- Diet *without* fatty fish was associated with an irregular pattern of physical activity and a gradual decrease in physical activity during winter.
- Diet *with* fatty was associated with a regular and stable pattern of physical activity during winter.
- Regular fatty fish consumption seems to be a behavioral strategy with positive repercussions on other health behaviors such as levels of physical activity throughout winter.

Fatty fish consumption has been shown to have an impact on two levels: objective underlying biological mechanisms and subjective (self-reported) health and well-being (1, 2). However, little is known about the effects of fatty fish consumption at the *behavioral level.* Physical activity such as regular exercise is regarded as an important resilience enhancing treatment strategy (3). Studies have shown that regular exercise affects cognitive functioning and biological mechanisms such as heart rate (HR) and heart rate variability (HRV) (4, 5). HR and HRV are important indices of both physical and mental health (6, 7). Unfortunately, winter has been shown to cause a reduction in physical activity behavior (8). Research has shown that cessation of physical exercise like aerobic training causes a significant reduction in HRV after only 4 weeks (4). Physical inactivity is an important risk factor for noncommunicable diseases like coronary heart disease (9), but also mental health problems (10). As winter is associated with higher rates of mortality caused by cardiovascular events (11) and impairment in mental states (12, 13), it is important to identify strategies that can prevent a reduction in physical activity during winter.

Recent research demonstrated that a fatty fish intervention during winter with a forensic inpatient sample characterized by mental health problems (1) caused improvement in a number of health-related variables, such as increased HRV and reduced HR, reduced anxiety, and improved cognitive or executive functioning (1, 2, 14). Both HRV and executive functioning are important underlying mechanisms involved in self-regulation and behavior control (15). Moreover, after the intervention period, the Fish group receiving fatty fish regularly reported better daily functioning compared to the Control group receiving an alternative meal without fatty fish, but with the same nutritional value as they normally received (1).

Importantly, the Control group showed a significant increase in resting HR (2) in addition to poor stress resilience as they showed a sustained suppression in HRV during the post-stress period (16). Both HR and HRV have been shown to be associated with a risk for different diseases such as coronary heart disease, cardiovascular diseases as well as sudden cardiac death (6, 17). Thus, an increased HR and suppressed HRV may be critical, especially in vulnerable people.

Grant and colleagues (11) found that the most important risk factor for the high death rate during winter seems to be vitamin D deficiency. In this regard, it can also be mentioned that in the Hansen and colleagues’ study (1) both groups had a significant decrease in vitamin D status from pre- to post-test (i.e. from summer to winter). At the start of the experiment, both groups had sufficient vitamin D levels (Fish group 85 nmol/L and Control group 75 nmol/L). However, by the end of the fatty fish intervention the level of vitamin D in the Fish group (71 nmol/L) was closer to the level regarded as optimal (US 75 nmol/L) (18) compared to the Control group (55 nmol/L). Since winter usually causes a reduction in the level of vitamin D (i.e. level of 25-hydroxy vitamin D) (19, 20), these findings were interesting as well. Importantly, vitamin D level was significantly related to some of the outcome variables influenced by the fatty fish consumption, such as daily functioning, sleep efficiency and HRV (1, 2). As the Control group in this study (1) showed a lower level of vitamin D status compared to the Fish group by the end of the intervention study, fatty fish consumption may therefore be a good compensatory strategy during winter, since it is a rich source of vitamin D (21).

Investigating the effects at the behavioral level will expand knowledge about health benefits of regular fatty fish consumption. Increased understanding of this domain will have important implications on health behavior and health promotion. Thus, the primary aim of this study was to explore the effects of diet at the behavior level, that is, levels of physical activity during winter. The secondary aim was to explore the relationship between vitamin D status and physical activity.

## Materials and methods

### Study design and participants

This study is one of a series of papers from a parallel group randomized controlled trial investigating the effects of a long-term fatty fish intervention on mental health (1, 2, 14, 16, 22). The intervention period took place in a secure facility between September 2008 and February 2009. The study protocol was approved by the Ethics Committee at Sand Ridge Secure Treatment Center (April 10, 2008), and it was in accordance with the Declaration of Helsinki and the US federal regulations. Participants were recruited by means of both written and oral information about the study. The time period for the recruitment process was April–May 2008. Participation was voluntary, and all candidates fulfilling the admission criterion (IQ > 75) were accepted. All participants signed an informed consent form, and they were informed about the option to withdraw from the study at any time for any given reason without penalty.

**Fig. 1:**
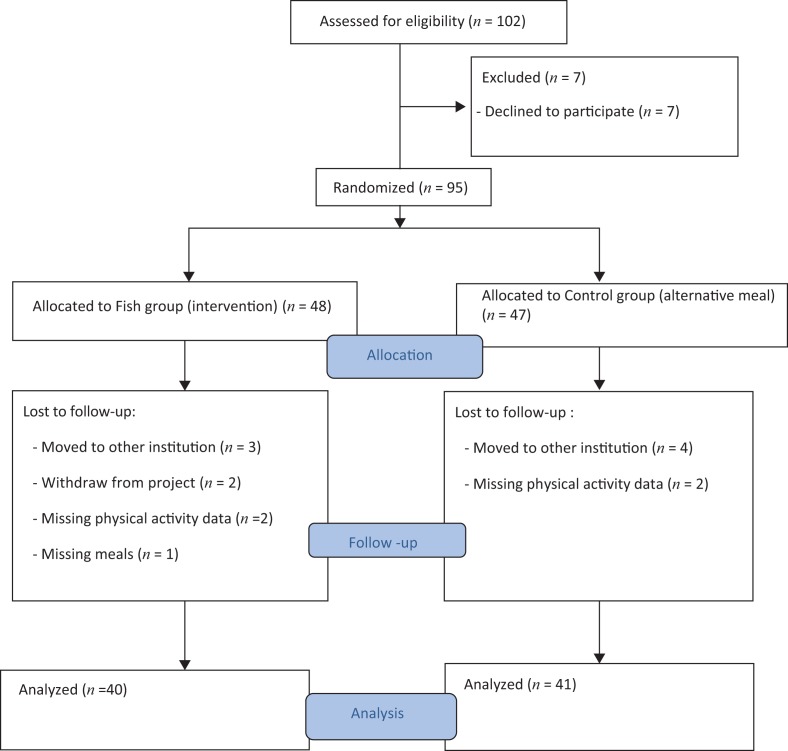
Flow of participants through this study.

Before randomization of the participants into Fish group or the Control group, the participants were matched on age, IQ (Wechsler Adult Intelligence Scale-Fourth Edition (WAIS-IV) (23) and Psychopathy Checklist List-Revised (PCL-R) score (24). For randomization, a computerized random number generator (in Excel) was used to assign each of the matched pairs. The random allocations to the groups were completed after all participants were enrolled and had completed baseline testing (pre-test battery). To balance the intervention (fish) and control groups (alternative meal) on age, IQ and PCL-R scores, stratified randomization was used. Random allocation was not concealed as the random allocation was done simultaneously for all subjects rather than sequentially. In order to match the participants, one of the co-authors enrolled participants and another co-author produced the matched pairs (stratification). The same person who enrolled the participants set up the implementation of the computerized random number generator which determined the group to which each participant would be assigned (2, 14). Figure 1, a CONSORT diagram (Consolidated Standards of Reporting Trials), presents the study progress for this particular study. As shown in the figure, 102 individuals were assessed for eligibility, of whom 7 declined to participate. Thus, a total of 95 participants were randomized to either the Fish group or the Control group. Due to different reasons 10 participants were lost to follow-up post-test (see Fig. 1). Another four participants (two participants in each group) did not complete registration of physical activity. Based on the risk of introducing a bias when replacing missing data (25), missing data were not replaced.

Thus, a total of 81 forensic inpatients with complex mental health problems (e.g. substance use disorders, personality disorders and/or affective disorders) participated in the study. Diagnoses were classified according to the Diagnostic and Statistical Manual of Mental Disorders (DSM-IV-TR) (cf. 14). Mean age was 42 years (range: 21–60). The total sample size was not determined by a power analysis performed prior to the experiment because of special circumstances in this intervention. The participants are a vulnerable group; therefore, all who met the inclusion criteria (IQ > 75) and were interested in joining were allowed to participate.

### Intervention

All participants in this study received some kind of food intervention – either fish or various kinds of meat meals, which were different from the routine institutional meals served. The Fish group received farmed Atlantic salmon (*Salmo salar* L.*)* for dinner three times per week from September to February. Originally, the intention was to serve the participants portion sizes of 200 g of salmon. The participants were used to larger dinner portions, so portions of 300 g were served for the main part of the intervention. During the last four weeks of the intervention portion, size was reduced to a more standard portion of 150 g three times per week. This was done to be able to carry out post-testing while the participants were still consuming fish, as the fish supply began to run low. When the Fish group received their fish, the Control group was provided an alternative meal of white or red meat (e.g. chicken, pork, beef). Importantly, this meal had the same nutritional value as they normally received, but it was different from the food the non-participating inpatients at the institution were served. This was done because blinding is impossible when it comes to food interventions. Thus, the Control group received meals consistent with the nutritional value of their habitual diet but still underwent an intervention. Compliance with meal consumption was monitored by the institutional staff. Participants were dismissed from the study if missing more than two meals in a month. Participants were offered a replacement meal when excused from a meal (e.g. unavailability at routine mealtime because of a medical appointment). According to the typical menu at the institution, tuna fish was served for dinner once a month, but the routine diet did not otherwise include fatty fish.

All food was prepared by the kitchen staff at the institution. The salmon was baked, prepared in a wok, or cooked as fish burgers. Different side dishes were used to provide a varied menu, and the two groups were served the same side dishes (e.g. vegetables, bread, and potatoes). The meals were repeated over a 12-week cycle. To determine nutrients and energy levels in the diet, double portions of all meals (i.e. breakfast, lunch, and dinner) during a week were collected and analyzed over 6 consecutive weeks during the intervention (cf. 16, 26). As it also has been reported before (cf. 16, 26), analysis of the nutritional composition of the diet showed that 100 g of the Control group’s diet contained energy 219 ± 22 kcal, protein 9.3 ± 1.0 g, vitamin D 2.9 ± 0.7 μg, sum EPA+DHA < 0.01 mg, and fat 13.3 ± 0.5 g. Moreover, for 100 g of the Fish group’s diet, the results showed the following: energy 198 ± 16 kcal, protein 9.1 ± 0.7 g, vitamin D 2.9 ± 0.7 μg, sum EPA+DHA 72 ± 6 mg, and fat 14.3 ± 1.0 g.

The salmon was analyzed for vitamin D content prior to shipping to the USA, and the results revealed that vitamin D content of the salmon overall ranged from 1.7 to 6.2 μg/100 g. Thus, one should expect that vitamin D content would be higher in the Fish group’s diet compared to the Control group’s diet. Importantly, from pre- to post-test the Control group also showed a higher drop in vitamin D status than the Fish group (cf. 1, 16). However, it should be noted that the double portions collected by the kitchen staff in the USA were collected over only 6 weeks due to financial concerns, as opposed to the whole 6-month period of intervention. The fish varies in vitamin D content, and the samples of double portions collected in the USA thus seem to be taken from fish with lower vitamin D content.

All diet samples were homogenized, freeze-dried, and pulverized before analysis of energy, total fat, protein, fatty acids, and vitamin D using accredited methods. Certified reference material was included in each run of the different nutrients. The trueness of each specific method has been tested by analyzing certified reference materials and by participation in proficiency test.

Other details concerning content of several undesirable substances in the fish have been described elsewhere (1).

### Outcome measure and test procedure

The participants completed the Physical Activity – Rating (PA-R) questionnaire developed by the National Aeronautics and Space Administration’s (NASA) Johnson Space Center (27) (also called the Physical Activity Status Scale [see 28] and University of Houston Non-Exercise Test for estimation of Maximum Oxygen Consumption [see 29]). The PA-R contains three main categories, which are further divided into seven subgroups. Listed in the order of increasing activity level, the main categories, subgroups, and their codes are as follows: Category 1) *No physical activity at all* (0 = no physical activity at all and 1 = physical activity such as go for a walk). Category 2) *Moderate physical activity or work such as jogging, ping pong/table tennis, resistance training, or hard physical work* (2 = 10–60 min per week, and 3 = more than 1 h each week). Category 3) *Regular hard physical activity such as running or jogging (treadmill), spinning or other forms of aerobic training* (4 = running, spinning or other such activities less than 30 min per week, 5 = running, spinning or other such activities 1.6–8 km per week or 1–3 h per week, 6 = running, spinning or other such activities 8–16 km per week or 1–3 h, and 7 = running, spinning or other such activities more than 16 km per week or more than 3 h per week). During the whole intervention period (September 2008–February 2009), the participants reported weekly the frequency, duration, and type of physical activity undertaken. This was done by filling in the proper code in a form.

### Statistical analyses

To describe the basic features of this data set we calculated weekly means and standard deviations for physical activity throughout the intervention for both groups. Additionally, standard deviations were calculated for each individual’s training score over the test period in order to take a closer look at the variability of each group. A *t*-test was used to test whether there was a significant difference between the groups (*α* < 0.05). The number of participants from each group in the upper quartile (higher degree of irregular exercise pattern) was identified, and the Mann-Whitney U test was used to test for statistically significant differences (*α* < 0.05). To investigate changes in physical activity during the intervention period, we used Repeated Measures Analysis of Variance (ANOVA), with two groups, that is, Fish versus Control group, as independent variables and physical activity per week as the dependent variable. The significant interaction was followed up by Fisher’s Least Significant Difference (LSD) test, but due to multiple time points and the risk of false positive findings (Type 1 error) the threshold was set to *α* = 0.001 for the follow-up test. For significant differences, effect sizes were calculated to investigate the magnitude of the differences (30). Finally, a mean score of physical activity was further analyzed in relation to vitamin D status with Pearson Product Correlations (*α* < 0.05).

## Results

### Descriptive statistics

Means and standard deviations for physical activity throughout the intervention period for the Fish and the Control groups are presented in Table 1.

**Table 1:** Means and standard deviations for physical activity throughout the intervention period for the Fish group and the Control group

Week	Control group (*n* = 41) *Mean* (*Standard Deviation*)	Fish group (*n* = 40) *Mean* (*Standard Deviation*)
1	3.56 (2.30)	4.03(2.20)
2	3.37(2.33)	4.28(2.15)
3	3.32(2.34)	4.18(2.23)
4	3.71(2.09)	4.13(2.08)
5	3.66(2.22)	4.03(2.20)
6	3.73(2.26)	3.98(2.15)
7	3.83(2.13)	3.98(2.22)
8	3.66(2.10)	3.9(2.17)
9	3.63(2.20)	3.95(2.04)
10	3.76(2.19)	4.08(2.00)
11	3.66(2.22)	4.05(1.97)
12	3.46(2.19)	3.95(2.09)
13	3.44(2.17)	4(2.09)
14	3.59(2.19)	3.93(2.15)
15	3.59(2.19)	3.88(2.11)
16	3.59(2.19)	3.95(2.07)
17	3.39(2.39)	4.15(2.20)
18	3.32(2.27)	4.18(2.18)
19	3.51(2.20)	4.1(2.11)
20	3.39(2.17)	4.03(2.06)
21	3.24(2.05)	4.08(2.08)
22	3.24(2.06)	4.15(2.02)
23	3.12(2.15)	4.1(2.02)
24	3.22(2.13)	4(2.04)
Mean	3.50(2.00)	4.04(2.02)

**Fig. 2:**
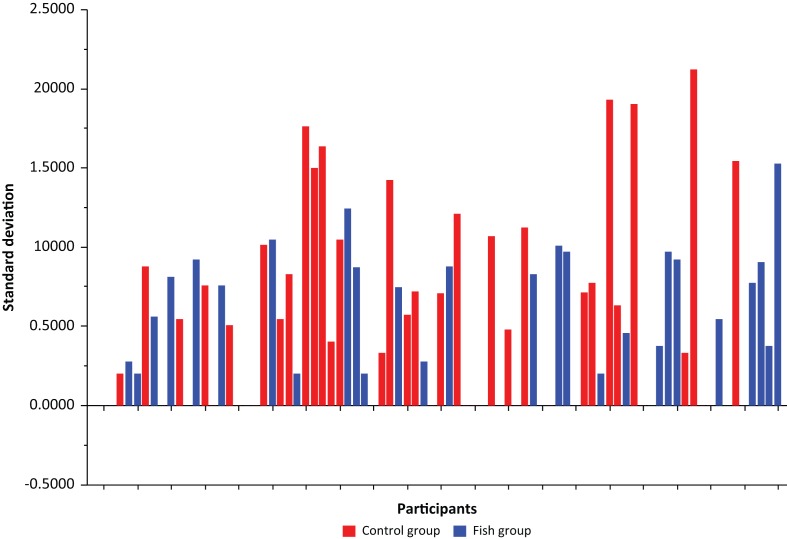
Standard deviation of the weekly physical activity. Red bars indicate the Control groups, blue bars indicate the Fish group.

A closer look at the standard deviations for each individual’s training score over the test period, that is, a *t*-test of the two groups, indicates a difference bordering on significance *t*(79) = −1.99, *P* = 0.050 (Fish group: *M* = 0.47, *SD* = 0.44; Control group: *M* = 0.72, *SD* = 0.63; *d* = 0.47). The bar graph in Figure 2 displays the standard deviation of the weekly self-reported physical activity patterns. Red bars designate members of the Control group, and blue bars indicate the Fish group. Nearly all the larger standard deviations, including the six largest, belong to those in the Control group, indicating a more irregular physical activity pattern in this group. Fifty-seven participants had variations in their physical activity pattern, of which 27 were in the Fish group and 30 in the Control group. An interesting pattern emerged when studying the upper quartile of this subsample of 57 participants. No less than 12 of the 15 subjects in this quartile belong to the Control group, and only 3 belong to the Fish group. Two of the participants, one from each group, had the same standard deviation, so there were 15 and not 14 participants in the upper quartile. The Mann-Whitney U-test showed that these values differed significantly, and that the number of participants in the groups was significantly different (*P* = 0.012).

### Diet and physical activity

**Fig. 3:**
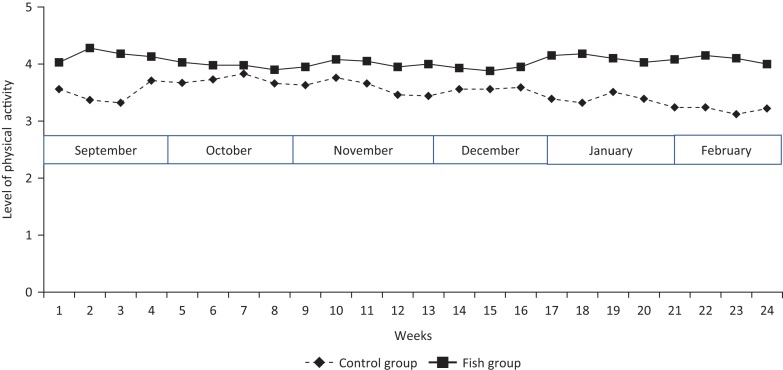
Levels of physical activity (means per week) during the whole intervention period (September–February) for the Fish group and the Control (meat) group.

Repeated measures ANOVA revealed no significant effect of groups or time (pre-post) *F*(1,79) = 1.49, *P* = 0.225, and *F*(23,1817) = 0.897, *P* = 0.603, respectively. However, a significant interaction between physical activity and diet groups was found, *F*(23,1817) = 2.02, *P* = 0.003. Follow up with Fishers’ LSD test revealed that there was no within-group differences in the Fish group (all *P*’s > 0.167). Thus, the Fish group showed a regular and stable pattern of physical activity from week 1 to week 24 (see Fig. 3).

In contrast, the Control group showed a successive reduction in physical activity over time. That is, there was a significant reduction from week 4 to week 23 (*P* = 0.001; *d* = 0.28), from week 6 to week 23 (*P* < 0.001; *d* = 0.28), from week 7 to week 21 (*P* = 0.001; *d* = 0.28), 22 (*P* = 0.001; *d* = 0.28), 23 (*P* < 0.001; *d* = 0.33) and 24 (*P* < 0.001; *d* = 0.29), and finally from week 10 to week 23 (*P* < 0.001; *d* = 0.29) (Fig. 3).

Looking at between-group differences, the follow-up test revealed no significant differences between the groups. Follow up test revealed that the lowest p-value was *P* = 0.044, and this was toward the end of the intervention (i.e. week 23). This is not significant regarding our threshold of *P* ≤ 0.001, but the effect size was *d* = 0.47.

### Correlations

The correlation analyses showed no statistical significant relationship between physical activity (mean score for the whole intervention period) and vitamin D status at pre-test (*r* = 0.20, *P* = 0.068). However, at post-test there was a small, but statistically significant relationship (*r* = 0.23, *P* = 0.045).

## Discussion

This study demonstrated a significant interaction between diet and physical activity during winter. Overall, the Control group, receiving a diet without fatty fish, showed an irregular pattern of physical activity, while the Fish group showed a more regular and stable pattern of physical activity throughout the whole intervention period. Importantly, the Control group also showed a significant and successive decline in physical activity over time. Finally, there was a weak relationship between vitamin D level and physical activity at post-test.

The current results showing that a diet without fatty fish is associated with a reduction in physical activity over time is in line with previous investigations. As physical activity influence HR (5) and HRV (4), this result corresponds to the increased HR and suppressed HRV found in the Control group (on a non-fish diet) in Hansen et al. (2) and Hansen et al. (16), respectively. However, based on this study it is not possible to conclude that increased HR and suppressed HRV can be explained by the decreased physical activity alone. The Control group did not eat fatty fish, and a relationship between fatty fish consumption and a reduction in HR has also been found (cf. 2). Other studies have shown that physical activity combined with a traditional Mediterranean diet emphasizing fish over other foods such as red meat (31), is associated with health benefits beyond what is achieved from the Mediterranean diet or high levels of physical activity alone (see 32 for an overview). Thus, future randomized controlled studies should investigate the combined effects of physical activity and fatty fish consumption on HR and HRV.

A close relationship between diet and physical activity was also supported by the results showing that regular fatty fish consumption seemed to prevent a decline in physical activity during winter. This adds something significant to the literature showing that fatty fish consumption in addition to having beneficial effects on objective and subjective measures of mental health (cf. 1, 2, 14) also influences *behavior* – the target factor with regard to significant lifestyle changes, health promotion, and prevention of both physical and mental diseases. As HRV and executive functions are regarded as underlying indices of self-regulation, and executive functioning is involved in behavior control related to a range of habits in everyday life (7, 33, 34), the current result is especially important as it validates previous results (1, 2, 14).

Due to the stable activity found in the Fish group, together with the successive decline in physical activity found in the Control group, there was a tendency toward an increasing difference between the groups toward the end of the intervention (see Fig. 3). As this study investigated the effects of a long-term intervention of 6 months, this pattern is of particular importance. On the follow-up of the significant interaction effect, the differences between the groups did not reach a statistically significant level at *P* = 0.001. The strongest Fishers’ LSD test for between-group differences revealed a *P* = 0.044 toward the end of the intervention (week 23). Although this difference was not statistically significant based on our threshold, the effect size was close to medium (*d* = 0.47), which is far from trivial (35). This complements the findings of Hansen et al. (1) showing that the Fish group reported better daily functioning compared to the Control group by the end of the intervention period. However, in Hansen et al.’s study (1) daily functioning was measured by a sleep diary, and the participants had to rate how they *felt* during daytime the day before. The PA-R (27) used in the present study is a measure of weekly physical activity behavior and exercise levels. As shown in Table 1, the mean scores for the Control group during the last 5 weeks of the intervention period (weeks 20–24) was close to Category 2 (mean score ≈ 3, i.e. moderate physical activity or work such as jogging, ping pong/table tennis, resistance training, or hard physical work). The mean scores for the Fish group throughout the whole period belongs to Category 3 (mean score ≈ 4, i.e. regular hard physical activity such as running or jogging [treadmill], spinning or other forms of aerobic training). This category involves physical exercise, a subclassification of physical activity. Mandolesi et al. (36) emphasized an important distinction between physical activity and physical exercise made by the World Health Organization (37). *Physical activity* could be any daily behavior or activity, while *physical exercise* requires something more, such as planning, structure, and repetition, as well as a precise frequency, duration, and intensity. Also this observation is interesting in light of the results showing that fatty fish consumption improved executive functions such as *planning* and *decision making* (cf. 14).

There may be several explanations of these results. A closer look at the descriptive statistics of the standard deviations showed that the Control group revealed a more irregular pattern of physical activity compared to the Fish group (Fig. 2). This complements the results from the ANOVA (Fig. 3) demonstrating that the Control group had a significant and successive decline in physical activity over time. There were no statistically significant between-group differences or within-group differences at the beginning of the study, but looking at Fig. 3 the repeated measures of weekly physical activity during the whole intervention period illustrates this irregularity in the Control group already from about the second week of the intervention. Based on this pattern of results there is reason to believe that a fish meal compared to a meat meal has immediate effects on both psychological and physical well-being and therefore increases the motivation to engage in physical activity and exercise. An important factor in this regard may be the time it takes to digest a fish meal compared to a meat meal. An anecdote in this respect has been that red meet such as beef is more filling compared to fish. However, there is evidence suggesting that a fish meal has a greater satiety compared to both chicken and red meat, and that it actually might take longer time to digest fish compared to chicken and meat (38). Comparing the immediate effects of consumption of red meat, chicken and fish, Uhe and colleges (38) found that fish consumption differed significantly from red meat and chicken on a number of biological factors. The decline in tryptophan:LNAA ratio was slower after a fish meal compared to red meat and chicken. The fish meal caused longer time for the amino acids to reach peak plasma concentration, and the plasma taurine and plasma methionine concentrations were greater after a fish meal. Thus, it was suggested that fish consumption might be associated with serotonergic activity (38). Importantly, serotonin has shown to vary throughout the year, and it has been argued that serotonin is important for energy balance, and that energy loss and fatigue during winter might be related to this variation (39). Thus, based on these previous results there seems to be evidence for significant differences in the immediate effects of fish versus meat consumption, which might explain the stable versus irregular pattern of physical activity in the fish versus control group, respectively. In the long run, the beneficial effects of fatty fish consumption could also be related to specific nutrients or interaction effects among different nutrients. As the present study showed a weak relationship between vitamin D status and physical activity, one may speculate whether vitamin D is important for mobilization of motivation and energy to engage in physical activity during winter as well. Interestingly, vitamin D is also important for serotonin (40). However, conclusions about the importance of serotonin cannot be made based on the current study. To gain in-depth knowledge about underlying mechanisms involved in the diet–physical activity interaction, more research is needed.

This study has both limitations and strengths that should be mentioned. One important limitation is the use of a subjective measurement of physical activity instead of an objective measure of physical fitness such as accelerometry or doubly labeled water (DLW) technique. Importantly, the DLW technique is regarded as the gold standard (41). However, this would have required other equipment and resources. Another limitation is the lack of a baseline measure as this study used physical activity as independent variable throughout the *intervention* period. Moreover, this study is limited by small group sizes, and only male forensic inpatients participated. Thus, we do not know whether the results can be generalized to other populations. What is striking with the current study is that the inpatients’ habitual diet consisted of mainly meat. Thus, the intervention truly did significantly change the diet of the Fish group, as they were unaccustomed to fish prior to the intervention. As the study was carried out in a forensic institution in the USA future studies should be conducted in other countries and in other settings or populations. Further investigation should also be done with larger groups and with both genders in order to draw stronger conclusions. Moreover, this study did not investigate the combined effect of physical activity and increased fatty fish consumption on other outcomes. Future studies should investigate the effects of four different conditions such as physical activity and fatty fish consumption in combination, fatty fish consumption only, physical activity only, and control condition, on physiological measures such as HRV and HR. However, in spite of these limitations the present study has important strengths. The sample included in this study is a relatively homogenous group. This can be regarded a strength, as physical activity has been shown to be related to socioeconomic position (42). This effect does not mask the findings of this study. Furthermore, as food interventions studies are difficult to perform successfully, this study is rare. It is a randomized control trial, and both groups received ‘special treatment’ as both groups received meals different from the usual institutional fare thrice weekly. We want to emphasize that the intention was not to feed the Control group with unhealthy food. The nutritional value of the control diet was the same as what they usually received at the institution (cf. 1, 16, 26). This study suggests that fatty fish should be served more frequently as it has impact on the behavioral level. Thus, this study has important implications for clinical practice, health behavior, and health promotion.

## Conclusion

Overall, the results of this study showed that type of diet throughout winter influenced the levels of physical activity. To our knowledge this is the first randomized intervention study investigating the effects of food intervention in relation to behavior such as physical activity. Importantly, the present study indicates that regular fatty fish consumption has positive repercussions on levels of physical activity throughout winter among men, while a diet without fatty fish tended to cause a negative effect. While vitamin D may be of importance for this effect, more investigation is needed in order to gain in-depth knowledge about the mechanisms of action.
